# Detection of respiratory inflammation biomarkers in non-processed exhaled breath condensate samples using reduced graphene oxide

**DOI:** 10.1039/d2ra05764f

**Published:** 2022-12-13

**Authors:** Azam Gholizadeh, Kathleen Black, Howard Kipen, Robert Laumbach, Andrew Gow, Clifford Weisel, Mehdi Javanmard

**Affiliations:** Department of Electrical and Computer Engineering, Rutgers University Piscataway NJ 08854 USA Mehdi.javanmard@rutgers.edu; Environmental Occupational Health Sciences Institute, Rutgers University Piscataway NJ 08854 USA; Ernest Mario School of Pharmacy, Rutgers University Piscataway NJ 08854 USA

## Abstract

In this work, we studied several important parameters regarding the standardization of a portable sensor of nitrite, a key biomarker of inflammation in the respiratory tract in untreated EBC samples. The storage of the EBC samples and electrical properties of both EBC samples and the sensor as main standardization parameters were investigated. The sensor performance was performed using differential pulse voltammetry (DPV) in a standard nitrite solution and untreated EBC samples. The storage effect was monitored by comparing sensor data of fresh and stored samples for one month at −80 °C. Results show, on average, a 20 percent reduction of peak current for stored solutions. The sensor's performance was compared with a previous EBC nitrite sensor and chemiluminescence method. The results demonstrate a good correlation between the present sensor and chemiluminescence for low nitrite concentrations in untreated EBC samples. The electrical behavior of the sensor and electrical variation between EBC samples were characterized using methods such as noise analysis, electrochemical impedance spectroscopy (EIS), electrical impedance (EI), and voltage shift. Data show that reduced graphene oxide (rGO) has lower electrical noise and a higher electron transfer rate regarding nitrite detection. Also, a voltage shift can be applied to calibrate the data based on the electrical variation between different EBC samples. This result makes it easy to calibrate the electrical difference between EBC samples and have a more reproducible portable chip design without using bulky EI instruments. This work helps detect nitrite in untreated and pure EBC samples and evaluates critical analytical EBC properties essential for developing portable and on-site point-of-care sensors.

## Abstract 2

In this work, we studied several important parameters regarding the standardization of a portable sensor of nitrite, a key biomarker of inflammation in the respiratory tract in untreated EBC samples.

## Graphical abstract

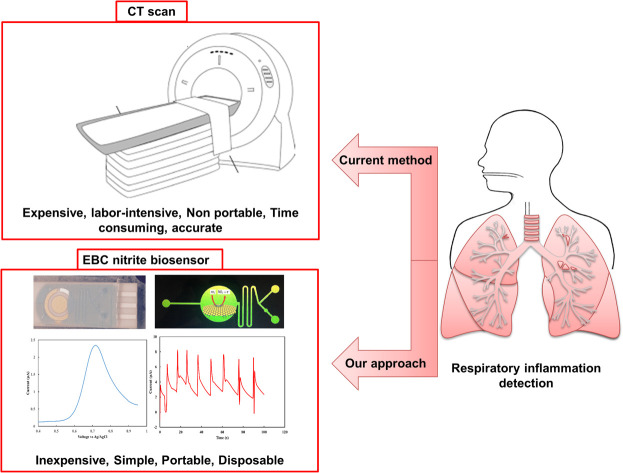

## 1. Introduction

Effective diagnosis and treatment of disease relies on accurate molecular tools that reflect a patient's clinical status. Development of new biomarkers and translating them into useful clinical tools requires novel applications of technology allowing point of care implementation.

Monitoring changes in airway inflammation in conditions such as asthma and obstructive pulmonary disease (COPD) can be useful for monitoring disease status and response to therapy. Exhaled breath condensate (EBC) is a matrix that is full of valuable biomolecular information. It is an attractive and non-invasive way to collect soluble components from the lower airways in the respiratory system. The correlation between exhaled breath condensate (EBC) biomarkers and pulmonary inflammation has been widely reported.^1–3^ Also, EBC is a promising alternative method to sampling of the inner lining of the respiratory airway compared to invasive methods to screen and early detection of diseases such as lung cancer.^4^ EBC is a liquid phase of the exhaled breath containing aerosol particles from the lower respiratory tract and water droplets that contains numerous numbers of biomarkers. For example, hydrogen peroxide and nitric oxide metabolites show increase in asthma,^5–7^ hydrogen cyanide can be detected in CF patients,^8,9^ 8-isoprostane can be a marker of oxidative stress in asthma and CF,^10–13^ and pH tends to be lower in severe asthma.^14,15^ The most acceptable clinical marker of airway inflammation in clinical practice is fractional exhaled nitric oxide (FeNO). Nitric oxide is a free radical gas generated from l-arginine, involving expression of one of the isoforms of nitric oxide synthases (NOS) called iNOS in airway epithelial cells.^16,17^ As the measured exhaled nitric oxide level is at the ppb level, extremely sensitive analyzers are needed. Nitric oxide analyzers are commercially available for clinical purposes, ranging from bulky and expensive chemiluminescence analyzers to hand-held devices relying on electrochemical technology. Although FeNO is commonly used for diagnosis purposes, nitrite oxide is a fleeting unstable molecule and is limited in sensitivity and specificity. Exhaled breath condensate (EBC) nitrite and nitrate are alternative byproducts of NO metabolism which have clinical or research significance.^18–23^ These molecules can be produced from nitric oxide with the reaction of free oxygen radicals in the respiratory system. As they are less volatile than nitric oxide and are in the liquid phase instead of gas, they are very promising biomarkers for assessing airway inflammation.

Several methods such as the Griess reaction, photoluminescence, and electrochemistry have been used to detect nitrite in EBC samples.^21,23–29^ All of these methods exhibit high sensitivity; however, they are non-portable and require sample pretreatment. The Griess reaction involves indirectly detecting nitrite by measuring the reaction between nitrite and aromatic amine. This reaction produces a purple azo dye. Since it can be prepared quickly, it is a widespread method. However, this method has some drawbacks for biological fluid analysis, thus producing unreliable results. The main problem is competing of other biomolecules present in the biological fluid with the nitrite reaction.^30^ This problem, coupled with a low concentration of nitrite in EBC samples, demands novel sensor materials and sensing modalities. In contrast, electrochemical methods are more sensitive, faster, require little pretreatment of samples, portable, simple design and low cost.^31^

We selected nitrite measurement as a biomarker of respiratory inflammation. Nitrite is electroactive with numerous electrode materials. However, it easily can foul the electrode surface thereby decreasing the sensitivity and accuracy.^32^ Various electrodes have been reported to overcome this issue, such as carbon nanotubes,^33^ carbon nanoparticles,^34^ metal nanoparticles,^35^ conductive polymers,^36^ enzymes,^37^ graphene,^38^ and various composite materials.^39^ The limit of detection in actual biological, environmental, and food samples for carbon nanomaterials has reached micromolar or even nanomolar levels, comparable with conventional methods.^40^ Among the carbon nanomaterials, graphene, a two-dimensional carbon material, has been widely used in electrochemical nitrite designs due to its unique electrochemical properties, including high surface to volume ratio, high electrical conductivity, wide electrochemical voltage range, and fast heterogeneous electron transfer.^41,42^ Several comprehensive reviews involving various nanofabricated graphene composite based nitrite sensors have been published, demonstrating the great potential of graphene in this analytical research area.^31,40,43^ Moreover, graphene-based electrochemical sensors have been applied to measure nitrite in biological samples, including work in human urine samples.^44–47^ A self-assembled polyoxometalate/GO/PDDA electrode on GCE was fabricated to measure nitrite in human blood serum with a detection limit of 0.45 μM.^48^ The potassium-modified graphene electrode was applied to determine nitrite released from liver cancer and leukemia cells.^49^ Also, our group reported an electrochemical graphene-based sensor that can detect nitrite in the biological range in EBC samples.^29^ Since then, other groups attempted to develop different electrochemical graphene composites to facilitate non-invasive detection of nitrite in EBC samples.^23^ However, numerous challenges still need to be addressed to attain the level of accuracy required for clinical application. Unresolved issues with assaying EBC include standardization of the sample collection, storage, and analytical method validation. In electrochemical sensors and other kinds of nitrite detection methods in EBC samples, sensor variability is still a major challenge, despite efforts by several groups.^17,50–52^

In this study, the effect of storage and other important analytical parameters such as choice of electrolyte, the effect of the EBC matrix, EBC pH were studied. To study the effect of EBC storage on sensor measurements, nitrite in EBC samples freshly collected from subjects were measured and compared an aliquot of the samples frozen at −80 °C for one month. For validation of the method, the set of measurements were compared with chemiluminescence sensor results. Electrical properties of the EBC sample matrix were also studied with electrochemical impedance spectroscopy (EIS) and electrical impedance (EI). A good correlation was observed between EIS, EI, and the peak location in DPV measurements. These data suggest the utility of applying peak location to calibrate electrical properties in portable devices. Our particular interest in this work was the study of the storage effect and electrical properties of EBC on nitrite detection response.

As compared with traditional nitrite detection technology, *e.g.*, mass spectroscopy and chemiluminescence, this highly sensitive sensor is much more cost-effective and requires small volume of sample, thus holding great promise as portable respiratory inflammation monitors. This reduced graphene oxide (rGO) based sensor offers several advantages. The high sensitivity of this sensor allows it to detect nitrite in ambient conditions. Reduced graphene oxide is highly amenable for large-scale fabrication, providing a cost-effective method to monitoring lung inflammations. Also, this sensor's results show the capability of the measurement of nitrite in untreated and pure EBC samples that is essential in designing portable point of care devices.

## 2. Materials and methods

### 2.1. Materials

Graphene oxide stock solution (2 mg ml^−1^ dispersion in H_2_O), sodium nitrite, and all other chemicals were purchased from Sigma Aldrich. Screen-printed three electrodes, including carbon as the working and counter electrode and Ag/AgCl as the reference electrode were used (Metrohm, USA).

The EBC samples were collected from volunteers using an RTube™ (Respiratory Research, Inc.) and a Jaeger EcoScreen. With the RTube, each subject used a new and separately packaged device to eliminate any risk of infection transmission. With the EcoScreen each subject used a disposable mouthpiece. By having subjects breathe through a one-way valve into a cooled condenser for 15 minutes, 3 ml of EBC sample was collected for each subject. The condensate was partitioned and either analyzed immediately or frozen for the storage evaluation. For the storage effect study, the samples were frozen at −80 °C. All surfaces were triple-rinsed with DI water before collecting EBC. Blank samples were collected from rinsing the collection device two times with DI water. Two EBC sample series were used in this study. EBC set 1 includes five volunteers, two blank samples used for the sample storage effect, and EIS experiments. EBC set 2 consists three volunteers, three blank samples used for the electrical measurements, and evaluation of the sensor performance using the chemiluminescence method. IRB number for this study is Pro20160000353.

Electrochemical reduction of graphene oxide was performed under nitrogen purging. All solutions were prepared using DI water. The rest of the experiments were done in ambient conditions. All electrochemical and electrical results are the average of three times analysis of two separate sensor measurements.

### 2.2. Instruments

Electrochemical measurements were performed using a potentiostat (Gamry 600, Gamry Instruments, Pennsylvania, USA). The morphology of graphene oxide was characterized using field-emission scanning electron microscopy (SEM) (Zeiss Leo Field Emission SEM), and FT-Raman spectra (micro Raman Spectrometer, 532 nm excitation laser) was used to obtain Raman shift of the electrode surface. pH microprobe was used to measure the pH of the EBC samples. As a gold standard method to benchmark the performance of our sensor, chemiluminescence detection (NOA 280i, GE analytics, boulder) was used to justify nitrite detection ability of the fabricated sensor. The electrical impedance was measured using a commercial lock-in amplifier (Zurich Instrument HF2A, Zurich, Switzerland).

### 2.3. Fabrication of sensor

#### 2.3.1. Nitrite sensor fabrication

The rGO electrochemical sensor was prepared with the spin coating deposition method. A thin PDMS membrane was spin-coated onto the screen-printed electrode. This step was performed for two purposes. First, the PDMS protects the counter and reference electrodes from electrical shortage during deposition of GO solution. Second, it serves as a substrate to fabricate a microfluidic channel. A 10 : 1 ratio of Sylgard PDMS to the curing agent was mixed thoroughly to prepare the PDMS mask. After degassing, the PDMS thin film was coated in a two-step coating with 500 rpm for 5 seconds and 4000 rpm for 30 seconds on the screen-printed electrodes. After curing in 80 °C for 1 hour, an approximately 20 μm thick PDMS membrane was formed on top of the electrodes. The film of PDMS on top of the working electrode was manually removed and cleaned with oxygen plasma. Then the solution of GO was deposited on top of the working electrode with the same coating parameters. Afterward, the PDMS membrane was removed from the counter and reference electrodes and the contact pads. Then, GO was reduced electrochemically using the cyclic voltammetry method. The voltage was swept between −1.6 to 0 V in 0.3 M acetate buffer (pH 5) with the scan rate of the 25 mV s^−1^ for 30 scans under the continuous nitrogen gas purging. The platinum wire was used as the counter electrode for reduction to avoid damage to the screen-printed counter electrode during the reduction process.

#### 2.3.2. Impedance sensor fabrication

Gold microelectrodes were used to measure the electrical impedance of the EBC samples. A 100 nm gold layer on top of the 10 nm chromium thin film was deposited on glass with electron beam evaporation. Then microelectrodes were patterned with standard lithography. To fabricate the chamber for EBC samples, a 3 mm hole was punched on the PDMS substrate. Then PDMS layer was bonded on a glass slide containing gold microelectrodes using oxygen plasma treatment.

#### 2.3.3. Microfluidic based nitrite sensor fabrication

A microfluidic-based nitrite sensor based on rGO was designed to analyze low-volume EBC samples. The microfluidic channel was fabricated using standard photolithography with a SU8 mask. The microfluidic part contains two channels for analyte and buffer injection, mixer, and solution reservoir parts. The fabricated channel on PDMS was bonded to rGO sensor using oxygen plasma. To avoid degradation of rGO and contact pads during the oxygen plasma treatment, they were covered with a piece of PDMS.

## 3. Results and discussion

### 3.1. Characterization of rGO thin film

**Fig. 1:**
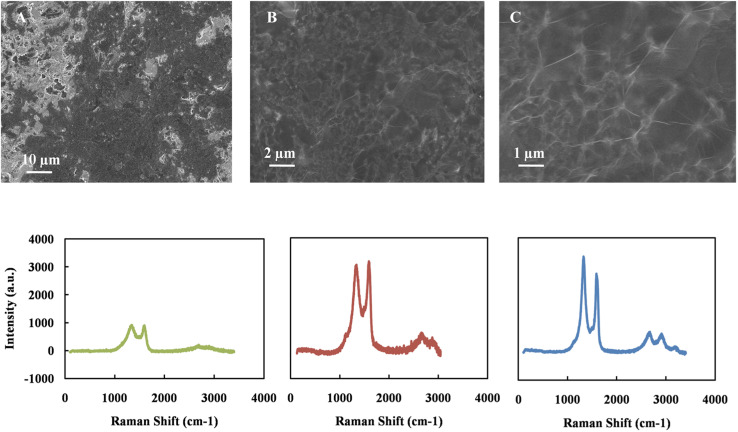
SEM of graphene oxide on carbon electrode in different magnifications, Raman spectroscopy of carbon working electrode (A), GO thin film (B) and rGO thin film (C) on top of carbon electrode.

The deposition method of GO is crucial to obtain good surface morphology, surface coverage, and film uniformity. The conventional methods to deposit GO for electrochemical sensors are drop-casting and dip coating.^53,54^ However, these methods often result in non-uniform deposition due to the aggregation of GO sheets and weak adhesion to the electrode surface. On the other hand, the solvent's rapid evaporation during spin coating produces a smoother surface with minimal wrinkling. This increases the adhesion between the GO thin film and the electrode surface and is critical to achieve reproducible results in electrochemical sensors, and during the reduction of GO sheets. Based on these advantages, spin coat was chosen to prepare GO thin film. Fig. 1 exhibits the spin-coated GO's SEM images on the working carbon electrode's surface with different magnification. The GO has covered most of the carbon electrode surface uniformly regardless of its micro-scale roughness. In order to evaluate the nature of the GO film, Raman spectroscopy was employed. Fig. 1 also shows the Raman spectrogram of bare carbon electrode surface (A), GO thin film (B), and rGO (C). The data represent the average of measurements that were recorded at different spots on each sample. Two peaks at around 1350 and 1580 correspond to the D and G bands, respectively. After reduction, the peak intensity ratio (*I*_D_/*I*_G_) increases from 0.9 to 1.2. This trend is attributed to the removal of the oxygen groups and decreases the domain size of the in-plane sp^2^ hybrids. This result concludes that the GO was reduced during the electrochemical reduction process.

### 3.2. Response of the sensor in blank EBC

**Fig. 2:**
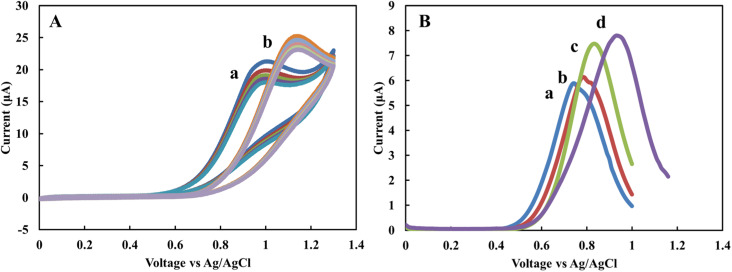
(A) Cyclic voltammetry of 1 mM nitrite in (a) 0.1 M acetate and (b) in 0.1 M PBS solutions. (B) Differential pulse voltammetry in (a) RTube blank, (b) EcoScreen blank, (c) 0.1 M acetate, (d) 0.1 M PBS buffer solutions. The scan rate is 50 mV s^−1^ for CV. DPV was performed with a step potential of 10 mV and pulse size 50 mV.

The EBC blank sample, as a supporting electrolyte, was investigated to evaluate the sensor's applicability for real-time measurements. The CV and DPV profiles of the sensor in the 1 mM standard nitrite solution were recorded and compared with acetate and PBS buffers. Fig. 2A shows CV of 1 mM nitrite solution prepared in acetate (a) and PBS buffers (b) for five scans with a scan rate of 50 mV s^−1^. Similar to previous results^29^ the nitrite peak in acetate appears at lower potentials in comparison to PBS. The variation of current for sequenced scans is 3.4 and 2 μA for acetate and PBS buffers, respectively. Fig. 2B shows DPV of 1 mM nitrite solution in the blank sample obtained from RTube (a) EcoScreen (b), 0.1 M acetate (c), and 0.1 M PBS buffer (d) solutions. The results show the electrode's current response for nitrite spiked in EBC blank samples is comparable with standard buffer solutions. This data indicates that EBC blank samples have enough electrical conductivity to be used directly as an electrolyte in electrochemical measurements in the portable biosensors.

**Fig. 3:**
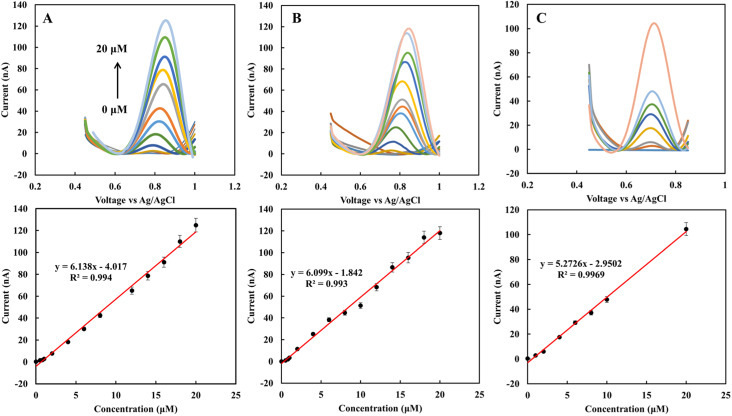
(A) Differential pulse voltammetry of different nitrite concentration at different nitrite concentrations of 0 to 20 μM spiked in RTtube EBC blank. (B) EcoScreen blank (C) 0.1 M acetate buffer. DPV was performed with a step potential of 10 mV and pulse size 50 mV.

To calculate the concentration of the nitrite in EBC samples, calibration curves were obtained using spiked nitrite solutions in the range of 0 to 20 μM in EBC blank samples with the DPV technique. Fig. 3A–C show the nitrite spiked calibration curve in RTube blank, EcoScreen blank solution, and 0.1 M acetate buffer, respectively. After removing the background measured signal and correcting for the baseline, calibration curves were obtained. The proposed sensor displays linear behavior in the biological range of nitrite in EBC samples from 250 nM to 20 mM. CV experiments of 5 mM K_3_[Fe(CN)_6_] in 0.1 M KCl were performed at various potential sweep rates (*ν*) to calculate the sensitivity of the sensor. A slope of 226.84 μA s^1/2^*ν*^−1/2^ (*R*^2^ = 1) resulted from *I*_p,a_*vs. ν*^1/2^. Based on the Randles–Sevcik equation with *n* = 1 and *D* = 7.6 × 10^−6^ cm s^−2^ for K_3_[Fe(CN)_6_] probe, the reduced graphene's effective surface area was estimated as 0.06 cm^2^. This calculation allows estimating the sensitivity of the nitrite sensor for different calibration curves in linear dynamic range. Sensitivity is 102.3, 101.65, and 87.87 nA μM^−1^ cm^−2^ for calibration curves of RTtube EBC blank, EcoScreen EBC blank, and 0.1 M acetate buffer, respectively. These results show better sensitivity for blank samples. It can be related to decreasing slight amount of nitrite in reaction with acetate buffer. The data prove blank samples as a good candidate for direct electrochemical determination of nitrite in EBC samples.

### 3.3. Storage effect on sensor response

**Fig. 4:**
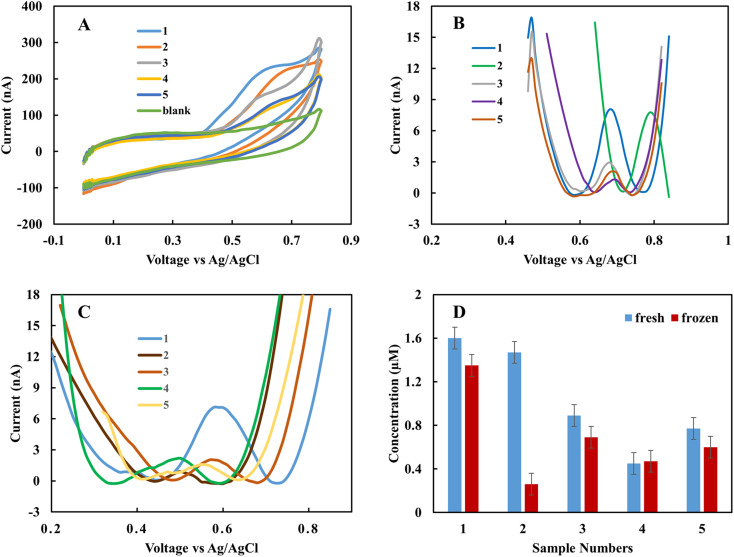
(A) CV of fresh EBC samples set 1 and the blank. (B) DPV of fresh EBC samples, (C) DPV of frozen EBC samples, (D) concentration of nitrite in fresh and frozen EBC samples. The scan rate is 50 mV s^−1^ for CV. DPV was also performed with a step potential of 10 mV and pulse size 50 mV.

**Fig. 5:**
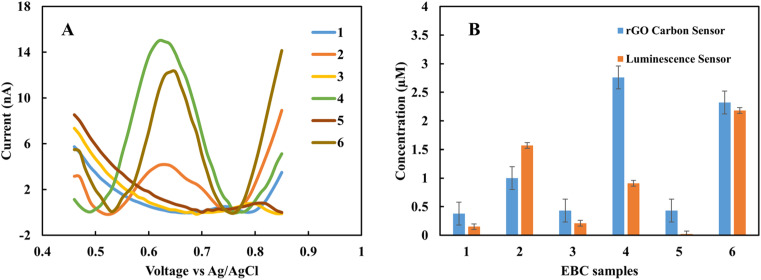
(A) DPV of EBC samples set 2 without pretreatment, (B) predicted nitrite concentration of pure EBC samples using rGO sensor and chemiluminescence.

In the clinical test, EBC samples were kept at −80 °C after collection from subjects. In this work, the storage effect on the sensor response was investigated. In one case study (EBC set 1), samples were collected from five subjects and measured immediately with the rGO sensor. Then the samples were kept at −80 °C for one month, and measurement was repeated. Each measurement was repeated three times with two different sensors, and the results are the average of these measurements. Fig. 4A shows the CV curves for fresh samples immediately after they were obtained from subjects. The results show that the fabricated sensor is sensitive to nitrite in complex biological samples, and in the applied range of voltage, there is no interference. The peak current occurs in the range of 0.68 to 0.78 V based on the individual samples' electrical nature. And the blank sample does not show any peak in the interested voltage area. For quantity analysis, DPV was used. Fig. 4B demonstrates the DPV results for fresh samples. Among five samples, two have a higher level of nitrite. The measurement was repeated one month later on frozen samples at −80 °C. Fig. 4C shows the achieved DPV curves. The concentrations were predicted from the calibration curve for each of the collection methods (Table 1). There is a good correlation between standard calibration curves peak location and EBC samples that provides a good selectivity for this sensor. For each collection method the corresponding predicted concentration was used. Among five samples, four of them show a diminishing amount of current for frozen samples (Fig. 4D). This variation is likely due to the conversion of nitrite into nitrate over time. These results suggest the importance of point of care and real-time electrochemical sensor designs for on-site study tools of pulmonary inflammation biomarkers such as nitrite. The performance of the sensor was compared with our previous nitrite sensor. Also, the chemiluminescence method was applied to evaluate the performance of the fabricated electrolyte-free electrochemical nitrite sensor for EBC samples. Fig. 5 shows the comparison between the rGO-gold sensor's performance that we developed previously^29^ and the rGO carbon-based sensor presented in this work.

**Table 1:** Predicted concentration of the fresh and frozen samples (EBC samples set 1)

Sample	Current (nA)	RTube method (μM)	EcoScreen method (μM)	Acetate method (μM)
**Fresh samples**				
1	8.58	1.60	1.96	2.07
2	7.79	1.47	1.81	1.92
3	3.08	0.63	0.89	0.96
4	2.08	0.45	0.70	0.76
5	2.38	0.50	0.77	0.82
**Frozen samples**				
1	7.10	1.35	1.68	1.78
2	0.89	0.26	0.46	0.52
3	2.04	0.45	0.69	0.75
4	2.18	0.47	0.72	0.78
5	1.58	0.36	0.60	0.65

Fig. 5A and B show the pure EBC samples' analysis without adding external electrolyte analyzed with the present sensor platform. These five samples were among the fifteen EBC samples containing blank and subject samples, and the sensor with 100 percent accuracy distinguished between blank and subject samples. While we could recognize nitrite in the μM range with our previous sensor, the new sensor shows good correlations with chemiluminescence results in lower range of nitrite concentration (<1 μM). Chemiluminescence analysis is same as we used in previous work.^29^ The *R*-squared of samples is 0.46% and as sample 4 as an outliner is 0.86%. The difference between some samples such as sample 4 can be related to the electrical properties of the individual subjects. The main difference between chemiluminescence and the electrochemical sensor is that the electrochemical sensor is sensitive to the electrical property of the sample matrix and ions concentration. One of this paper's main goals is to better understand this effect in untreated samples and use these data to calibrate the predicted concentration in the next step.

### 3.4. Electrical properties of the sensor

One of the important parameters in electrochemical sensors is the electrical behavior of the sensor and electrical conductivity of the individual subject's sample. In this work, electrochemical noise analysis and electrochemical impedance spectroscopy were used to study the electrical properties of rGO sensor. Also, electrical and electrochemical impedance were performed to study the electrical properties of EBC samples.

**Fig. 6:**
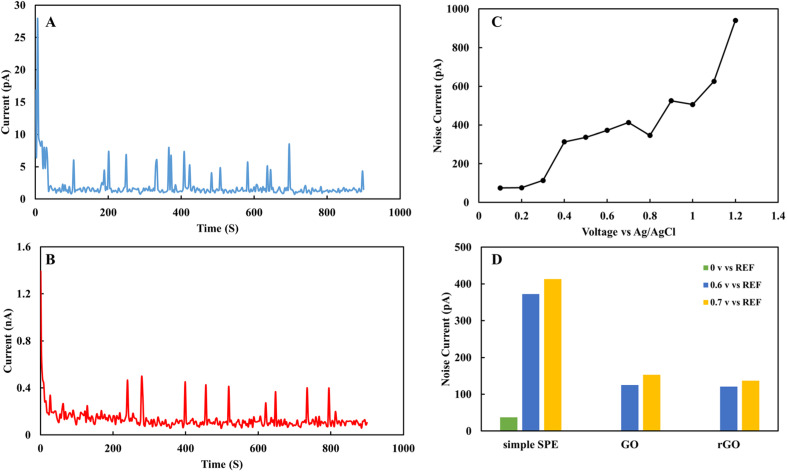
Electrochemical noise analysis of (A) rGO electrode in 0 V, (B) rGO electrode in 0.7 V, (C) SPE electrode respect to applied voltage range of 0 to 1 V, (D) average noise level for SPE, GO, and rGO electrode in 0, 0.6, and 0.7 V in 1 mM nitrite in 0.1 M acetate buffer solution. All voltages were applied *versus* Ag/AgCl reference electrode.

Fig. 6 shows the electrochemical noise analysis of the nitrite sensor. It was used to study signal to noise ratio in 1 mM nitrite in acetate solution. Fig. 6A demonstrates fifteen minutes noise level of rGO sensor in nitrite solution at zero voltage. Fig. 6B shows fifteen minutes noise level of rGO sensor in 0.7 V. Fig. 6C indicates variation of the average noise level respect to applied voltage of SPE in the voltage range of 0 to 1 V. Fig. 6D shows the average noise level for SPE, GO and rGO sensors in 0, 0.6 and 0.7 V. rGO based sensor shows noise level between 2 to 100 pA for 0 to 0.7 V and signal to noise ratio for rGO sensor is 7 μA/173 pA = 92 dB. Based on the overall data in 0.7 V that is the active peak voltage for nitrite, rGO shows the lowest noise level and more stable surface to analyze nitrite compared to SPE and GO sensors.

**Fig. 7:**
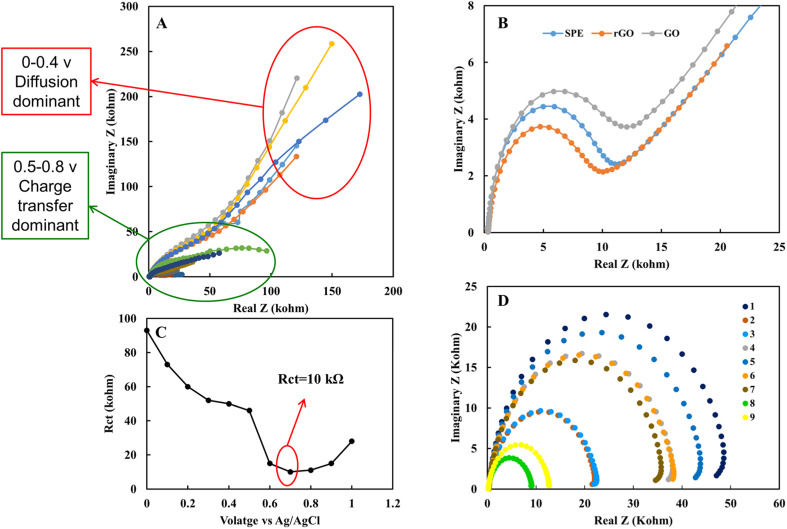
(A) Electrochemical impedance spectroscopy data of 1 mM nitrite solution in EBC blank sample on rGO electrode in the range of 0 to 8 DC bias voltage. (B) Electrochemical impedance spectroscopy data of 1 mM nitrite solution spiked in EBC blank sample on SPE, GO and rGO electrodes. (C) Simulated amount of charge transfer resistance for rGO electrode. (D) Electrochemical impedance spectroscopy data of EBC samples set 1 on rGO electrode. The spectrums were taken at 0.1 Hz to 1 MHz with AC potential amplitude is 10 mV.

Electrochemical impedance spectroscopy is another powerful tool to study the electrical properties of the electrode's surface and solution. Fig. 7A shows the EIS obtained in the voltage range of 0 to 8 volt DC bias in 1 mM nitrite solution in EBC blank sample on rGO electrode. Results show diffusion behavior in the range of 0–0.4 V. As the voltage keeps getting closer to the redox peak, the electrical behavior converted into the charge transfer one. The impedance behavior of SPE, GO and, rGO was compared. rGO shows the lowest, and GO shows the highest charge transfer resistance in 1 mM nitrite solution (Fig. 7B). The lowest charge transfer resistance for rGO electrode of 10 kohm occurs in 0.7 V is comparable with the calibration curves achieved with the DPV method (Fig. 7C). Also, the EIS of individual subject samples of EBC set 1 was investigated (Table 2). As it is evident from Fig. 7D, the electrochemical nature of EBC samples is different for individual subjects due to variation in ionic concentration of EBC samples. This result also emphasizes the importance of point of care medical sensor designs that can take into account the unique electrical properties of each biological sample type.

**Table 2:** Electrochemical impedance spectroscopy parameters for EBC samples (EBC samples set 1)

Sample	Type	*R*_ct_ (kohm)	*R*_s_ (ohm)	*C* (nF)
1	R-tube sample	49.2	279.4	748.3
2	R-tube sample	38.4	280.0	771.8
3	R-tube sample	22.1	279.2	821.0
4	EcoScreen sample	44.3	273.1	775.5
5	EcoScreen sample	22.4	275.7	762.2
6	EcoScreen blank	38.7	279.7	846.9
7	R-tube blank	36.0	286.4	812.3
8	0.1 M acetate	9.0	200.0	1044.0
9	0.1 M PBS	12.6	188.8	780.4

**Fig. 8:**
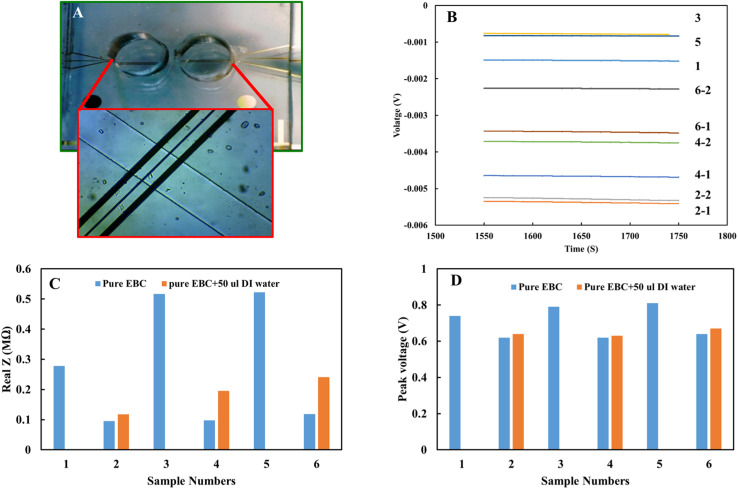
(A) Optical image of the gold microelectrodes, (B) electrical impedance results for EBC samples set 2 with 0.4 V applied voltage and 500 kHz frequency (sample 1 and sample 2 refer to pure and DI water added ones). (C) Comparing electrical impedance results for EBC samples with and without adding DI water, (D) voltage shift of DPV results for EBC samples.

The EIS analysis results indicate an electrical difference between the EBC samples and emphasize the ionic nature difference among the subject samples. This variation can affect the accuracy of the nitrite concentrations calculated from the standard calibration curve. Also, the electrical conductivity of the electrolyte in electrochemical measurements affects redox potential values. This variation was observed in the EBC sample analysis. This voltage variation can be a straightforward way to calibrate the results for individual biological samples regarding the nature of each sample's electrical properties. The pure electrical property of EBC samples from set 2 was measured with electrical impedance using gold microelectrodes to evaluate this idea. Fig. 8A shows the device was used to obtain electrical impedance data. To ensure accuracy of results, further analyses were performed in EBC samples with DI water added intentionally to reduce electrical conductivity. Fig. 8B and C show the impedance of the six different EBC samples, three of which had added DI water. Measurements took place by applying 0.4 V with a frequency of 500 kHz. Results show higher impedance for samples with DI water and the electrical impedance difference between individual EBC samples. Fig. 8D shows the overpotential obtained from the electrochemical analysis. Compared with the electrical impedance (Fig. 8C), there is a similar trend between overpotential and electrical impedance. These data interestingly indicate that overpotential can be used to calibrate the electrical properties of individual subject samples. pH of the individual EBC samples also was measured. The data did not show a significant difference among the samples and different collection methods (Table 3).

**Table 3:** pH of EBC samples using microelectrode pH meter (EBC samples set 1)

Sample	Type	pH
1	R-tube sample	7.89
2	R-tube sample	7.70
3	R-tube sample	7.64
4	EcoScreen sample	7.64
5	EcoScreen sample	7.67
6	EcoScreen blank	7.61
7	R-tube blank	7.68
8	0.1 M acetate	6.00
9	0.1 M PBS	7.00

**Fig. 9:**
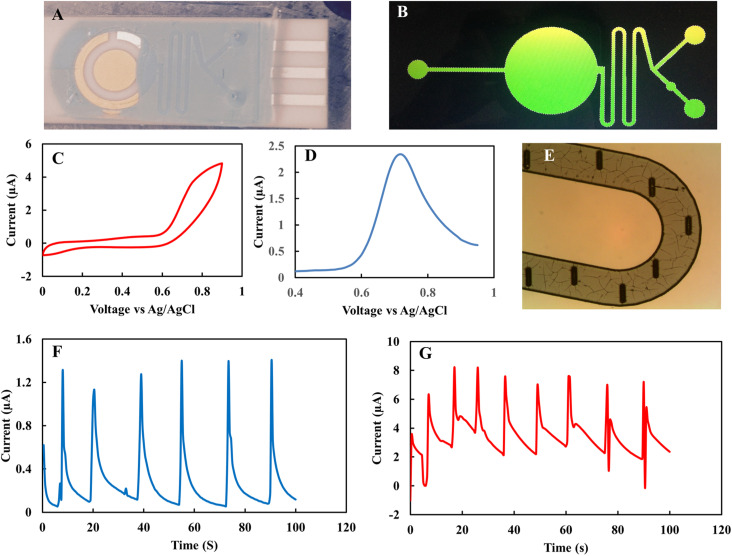
(A) Microfluidic based nitrite sensor, (B) schematic of the microfluidic channel, (C) CV of the 1 mM nitrite solution in 0.1 M acetate buffer. Scan rate 50 mV s^−1^. (D) DPV of the 1 mM nitrite solution in 0.1 M acetate buffer. (E) PDMS pillars inside the channel, (F) chronoamperometry of 5 mM K_3_Fe(CN)_6_ solution in 0.1 M KCl. (G) Chronoamperometry of 1 mM nitrite solution in 0.1 M acetate buffer.

As collection of EBC sample is time-consuming the microfluidic-based nitrite sensor was also investigated as a means to decrease the required volume of EBC sample per test to 10 μL. Fig. 9A and B show the microfluidic channel that was used on top of the nitrite sensor. By applying a thin layer of PDMS layer on top of the SPE electrode, the microfluidic channel layer can be easily integrated into the sensor. Fig. 9C and D show the CV and DPV response of the microfluidic sensor of the 1 mM nitrite solution. PDMS micro-pillars also were used as a mixing component in the mixer part (Fig. 9E). The chamber contained two inlets, one for continuous flow of buffer solution and another for injection of the analyte. Fig. 9F shows the CV and chronoamperometry responses of microfluidic nitrite rGO sensor in successive injections of the 5 mM K_3_(Fe[CN]_6_) in 0.1 M KCl standard solution. After confirming the electrochemical performance of the device with a standard solution, the 1 mM nitrite in 0.1 M acetate sample was characterized using the microfluidic channel. Fig. 9G shows the successive injection of nitrite analyte with a continuous flow of the buffer solution. The data shows promising repeatable responses from the microfluidic platform towards nitrite analyte. Further study is required to optimize the flow rate and its operation by integrating a microvalve into the sensor system to provide more accurate control of the concentration of the analyte in EBC samples.

## 4. Conclusion

In this work with pure EBC samples, we studied the response of reduced graphene label-free electrochemical sensors of nitrite, one of the key oxidative markers in respiratory inflammation and diseases. We have shown this sensor to be capable of detecting nitrite in pure EBC samples without any pretreatment. We demonstrated the feasibility of development of on-site portable respiratory biomarkers detection. Our data emphasize that storage time has an impact on the amount of nitrite quantified in EBC samples, thus demonstrating the importance of developing tools that can perform measurements on site. The sensor has high sensitivity in detecting nitrite below the nM concentration range with low sample volume requirements. Various electrical measurements were applied to investigate the electrical properties of both the electrode and the EBC samples. The shift in the redox voltage also indicates the potential for calibration of electrical properties of the individual samples. Significant variability in pH across samples, however, was not observed. Variability in EBC electrical phenotype among subjects was, however, observed thus emphasizing the importance of performing multiple measurements. Future work will be dedicated to developing multi-modal sensor platforms that can measure the relevant parameters in conjunction with the electrochemical measurement in order to build an accurate point of care detection apparatus and investigating cross reactivity. Another path forward would be collecting samples from healthy and unhealthy subjects and analyzing the sensor's performance. Also, in addition to the long-term storage effect, the time-dependent and thaw-cycling storage study can give valuable data regarding the portable device design.

## Conflicts of interest

The authors declare no conflict of interest.

## Supplementary Material
